# Phase 1 study of ARQ 761, a β-lapachone analogue that promotes NQO1-mediated programmed cancer cell necrosis

**DOI:** 10.1038/s41416-018-0278-4

**Published:** 2018-10-15

**Authors:** David E. Gerber, M. Shaalan Beg, Farjana Fattah, Arthur E. Frankel, Oluwatomilade Fatunde, Yull Arriaga, Jonathan E. Dowell, Ajit Bisen, Richard D. Leff, Claudia C. Meek, William C. Putnam, Raja Reddy Kallem, Indhumathy Subramaniyan, Ying Dong, Joyce Bolluyt, Venetia Sarode, Xin Luo, Yang Xie, Brian Schwartz, David A. Boothman

**Affiliations:** 1 Department of Internal Medicine (Division of Hematology-Oncology), University of Texas Southwestern Medical Center, Dallas, TX 75390 USA; 2 Department of Clinical Sciences, University of Texas Southwestern Medical Center, Dallas, TX 75390 USA; 3 Harold C. Simmons Comprehensive Cancer Center, University of Texas Southwestern Medical Center, Dallas, TX 75390 USA; 4 Texas Tech University Health Sciences Center School of Pharmacy, Dallas, TX 75390 USA; 5 Department of Pathology, University of Texas Southwestern Medical Center, Dallas, TX 75390 USA; 6 Department of Bioinformatics, University of Texas Southwestern Medical Center, Dallas, TX 75390 USA; 7 ArQule, Inc., Burlington, MA 01803 USA

## Abstract

**Background:**

NAD(P)H:quinone oxidoreductase 1 (NQO1) is a two-electron oxidoreductase expressed in multiple tumour types. ARQ 761 is a β-lapachone (β-lap) analogue that exploits the unique elevation of NQO1 found in solid tumours to cause tumour-specific cell death.

**Methods:**

We performed a 3+3 dose escalation study of 3 schedules (weekly, every other week, 2/3 weeks) of ARQ 761 in patients with refractory advanced solid tumours. Tumour tissue was analysed for NQO1 expression. After 20 patients were analysed, enrolment was restricted to patients with NQO1-high tumours (*H*-score ≥ 200).

**Results:**

A total of 42 patients were treated. Median number of prior lines of therapy was 4. Maximum tolerated dose was 390 mg/m^2^ as a 2-h infusion every other week. Dose-limiting toxicity was anaemia. The most common treatment-related adverse events were anaemia (79%), fatigue (45%), hypoxia (33%), nausea (17%), and vomiting (17%). Transient grade 3 hypoxia, reflecting possible methemoglobinaemia, occurred in 26% of patients. Among 32 evaluable patients, best response was stable disease (*n* = 12); 6 patients had tumour shrinkage. There was a trend towards improved efficacy in NQO1-high tumours (*P* = 0.06).

**Conclusions:**

ARQ 761 has modest single-agent activity, which appears associated with tumour NQO1 expression. Principal toxicities include anaemia and possible methemoglobinaemia.

## Introduction

Beta-lapachone (β-lap) is a quinone derived from the bark of South American Lapacho tree (*Handroanthus impetiginosus*) that exerts specific anti-tumour effects by capitalising on the increased concentration of NAD(P)H:quinone oxidoreductase 1 (NQO1) in cancer cells vs. normal cells. NQO1 is a two-electron oxidoreductase expressed in cancer tissue at levels 5- to 200-fold greater than in normal tissue. In cells overexpressing NQO1, β-lap initiates a futile redox cycle resulting in reactive oxygen species (ROS) generation.^1^ In turn, these ROS cause DNA single-strand breaks, hyperactivation of poly(ADP-ribose) polymerase-1 (PARP-1), loss of NAD+ and ATP pools, and a unique pattern of cell death referred to as “programmed necrosis” or “necroptosis”. Programmed necrosis has attributes of both apoptosis (e.g. terminal deoxynucleotidyltransferase-mediated dUTP nick-end labelling positive and chromatin and nuclear condensation) and necrosis (e.g. caspase and energy independent).^2^

In contrast to many molecularly targeted therapies, β-lap has the potential for broad anticancer activity. Compared to normal tissue, NQO1 overexpression occurs up to 200-fold in >80% of non-small cell lung cancer (NSCLC), up to 100-fold in >80% of pancreatic cancer, up to 10-fold in 60% of prostate cancer, up to 10-fold in 60% of breast cancer, and up to 10-fold in 50% of colorectal cancer.^3–6^ In preclinical models, β-lap-induced cancer cell death occurs across tumours in proportion to tumour NQO1 levels.^6–8^ In normal tissues, induction of NQO1, a phase 2 detoxifying enzyme, occurs rarely (e.g. with substantial exposure to polyaromatic hydrocarbons) and is short-lived (<2 h). Importantly, factors associated with ROS detoxification (and presumptively β-lap resistance) such as catalase expression are deficient in cancer cells relative to normal tissue, further enhancing the potential therapeutic margin of this strategy in specific cancers. β-Lap has also demonstrated synergy with ionising radiation, cytotoxic chemotherapy, and PARP inhibitors,^9,10^ rendering it potentially useful as a cancer therapy.

Despite a strong rationale and promising preclinical data, to date the clinical utility of NQO1-bioactivatable drugs has been limited. Early drugs in this class, such as mitomycin C and E09, which act by alkylating DNA, convey substantial toxicity and are susceptible to multiple resistance mechanisms, including apoptotic deficiencies and increased DNA repair.^11,12^ ARQ 501 (ArQule, Woburn, MA), a fully synthetic, insoluble form of β-lap that was formulated in hydroxylpropyl-β-cyclodextran (HPβCD), advanced to single-agent and combination clinical trials in multiple cancer types.^13,14^ Unexpectedly, haemolytic anaemia emerged as the predominant dose-limiting toxicity (DLT) of ARQ 501. Subsequently, preclinical models suggested that ARQ 501-associated haemolytic anaemia arose from complexation with HPβCD, the carrier required for solubility,^15^ rather than from β-lap alone.^16^ Furthermore, studies of ARQ 501 did not include assessment of NQO1 biology because the drug was being developed primarily as an E2F-targeting cell cycle inhibitor.^13,14,17^

ARQ 761 (ArQule, Burlington, MA) is a synthetic prodrug of β-lap that is more water soluble and requires <5% of the HPβCD required for ARQ 501 administration, thereby conveying a lower hypothetical risk of haemolytic anaemia. Upon dosing, ARQ 761 is rapidly and completely converted to ARQ 501. We conducted a first-in-human phase 1 dose escalation trial of ARQ 761 in refractory advanced solid tumours. Based on our preclinical findings and β-lap’s mechanism of action, we included assessment of tumour NQO1 expression by immunohistochemistry (IHC) as an exploratory (and subsequently enrolment) biomarker. We also analysed NQO1 genotypic polymorphisms, which are associated with the absence of NQO1 in normal tissue and may influence drug exposure,^18,19^ using a rapid blood assay.

## Methods

This clinical trial (NCT01502800) was approved by the University of Texas Southwestern Medical Center Institutional Review Board (IRB study number 042011–005). All subjects provided written informed consent prior to undergoing any study-related procedures. This was an open-label, single-institution, 3+3 dose escalation phase 1 trial, initially designed to include a planned biomarker-selected expansion cohort at the maximum tolerated dose (MTD). The primary objective of the study was to determine the safety, tolerability, and MTD of ARQ 761. Secondary objectives included efficacy and pharmacokinetic (PK) analyses. Exploratory objectives included predictive and pharmacodynamic biomarkers.

### Patient selection

Key inclusion criteria included the following: refractory advanced solid tumour for which standard curative or palliative measures do not exist or are no longer effective; ≥4 weeks since prior chemotherapy, ≥3 weeks since prior radiation therapy, ≥2 weeks since prior molecularly targeted therapy (including small molecules and monoclonal antibodies); measurable disease by Response Evaluation Criteria in Solid Tumours (RECIST) version 1.1;^20^ Eastern Cooperative Oncology Group performance status 0–1; central venous access (due to local haemorrhage and infusion site necrosis upon repeated dosing in preclinical studies and intolerance of repeated peripheral dosing of ARQ 501 in earlier clinical trials); adequate clinical laboratory parameters (absolute neutrophil count [ANC] ≥ 1500/µL, haemoglobin ≥ 10 g/dL [transfusion permitted up to 7 days prior to enrolment], platelets ≥100,000/µL, total bilirubin less than institutional upper limit of normal [ULN], aspartate aminotransferase and alanine transaminase ≤2.5 × institutional ULN [≤5 × institutional ULN in the presence of liver metastases], creatinine clearance ≥60 mL/min/1.73 m^2^); and availability of 10 unstained slides or paraffin-embedded tissue block from archived tumour specimen. After analysis of the first 20 patients with available efficacy and tissue biomarker data, enrolment was restricted to patients with NQO1-positive tumours (defined as *H*-score ≥200). We permitted tissue pre-screening, such that patients’ NQO1 status could be determined prior to their needing initiation of new treatment. Principal exclusion criteria were untreated brain metastases, leptomeningeal disease of treatment status, pregnancy, and concurrent receipt of hepatic enzyme-inducing antiseizure drugs. There was no limit to the number of prior cancer therapies.

### Study treatment

Initially, ARQ 761 monotherapy was administered as a 1-h weekly infusion. During the conduct of the trial, the emergence of frequent and clinically significant haemolytic anaemia at higher doses led to changes in infusion length and schedule, with ARQ 761 administered as a 2- or 3-h infusion weekly, every other week (QOW), or 2/3 weeks. Prolongation of infusion duration was implemented after we observed more haemolytic anaemia with the 1-h 390 mg/m^2^ dosing level than we did with the 195 mg/m^2^ dose. However, in a single patient treated at the 390 mg/m^2^ dose, we noted evidence of early recovery from haemolysis despite ongoing weekly dosing, when the infusion duration was prolonged from 1 to 2 h due to a suspected mild infusion-related reaction. This observation persisted within the expansion cohort of 195 mg/m^2^ dose level, for which haemoglobin levels were more stable level for patients who received 2-h infusions than those for those who received 1-h infusions. ARQ 761 was administered without premedication. Treatment was continued until disease progression or unacceptable toxicity.

The starting dose of 195 mg/m^2^ represented 50% of the MTD of ARQ 501, the earlier, insoluble formulation of β-lap that was associated with DLT attributed to high levels of the carrier molecule HPβCD. The dose was increased by 100% after the first dose cohort, then by 40% after subsequent cohorts. Intra-patient escalation from lower dose levels to successfully administered dose levels was permitted. DLT was defined as any of the following toxicities considered possibly, probably, or definitely related to ARQ 761 during the first cycle (4 weeks for weekly or bi-weekly regimen; 3 weeks for 2/3 weeks regimen): (1) ≥grade 3 haemolysis or haemolytic anaemia (transfusion for haemolysis or haemolytic anaemia was considered a DLT only if haemoglobin <8.0 g/dL [threshold for grade 3 anaemia]); (2) grade 4 neutropenia or thrombocytopenia, or grade 3 thrombocytopenia in the presence of bleeding; and (3) ≥grade 3 non-haematological toxicity that does not spontaneously resolve within 2 h post-infusion. Grade 3 nausea/vomiting or diarrhoea was considered a DLT only if it occurred despite optimal medical management. Grade 3–4 hyperbilirubinaemia was considered a DLT only if it did not recover to grade 1 after 7 days. When transient grade 3 hypoxia was observed at the completion of infusion based on pulse oximetry (a surrogate marker of methemoglobinaemia) without clinically significant symptoms, it was not considered a DLT. Dose reductions were implemented for recurrent grade 3 and any grade 4 non-haematologic toxicities.

### Study assessments

Safety assessments consisted of monitoring patient-reported symptoms, pre- and post-infusion vital signs, physical examination findings, blood and urine tests, and ECGs. In addition to standard blood counts and chemistries, markers of haemolysis (reticulocyte count, haptoglobin) were collected regularly. Toxicities were graded according to the NCI CTCAE, Version 4.0. Patients were assessed for response to therapy using RECIST 1.1 every 2 cycles of therapy. In some patients with hypoxia noted on pulse oximetry suggestive of methemoglobinaemia, arterial blood gas was obtained. In the event of a change in haemoglobin related to ARQ 761 therapy, the following tests could be performed: peripheral blood film, Coomb’s test (direct and indirect), reticulocyte count, haptoglobin, methemoglobin, bilirubin (total and direct), lactate dehydrogenase (LDH), free plasma haemoglobin, and oxygen saturation. Subjects were eligible to continue treatment if neutrophil count was ≥1000/mL, platelets were ≥50,000/mL, and haemoglobin ≥8.0 mg/dL.

### PK studies

Because ARQ 761 is rapidly and completely converted to ARQ 501 upon dosing, we estimated the PKs of ARQ 761 by following the plasma concentrations of ARQ 501 (β-lap). During the first and fourth infusions of study drug, serial blood samples for determination of plasma ARQ 501 levels were drawn at the following time points: pre-infusion, infusion start, 15 min, 30 min, 60 min, 120 min (for the 2-h infusion cohort), 15 min post-infusion, 1 h post-infusion, 3 h post-infusion, 5 h post-infusion, 24 h post-infusion, 48 h post-infusion, and 168 h post-infusion. ARQ 501 plasma concentrations were determined using an ultra-high-performance liquid chromatography tandem mass spectrometric analytical method. This method utilised a Nexera Series UHPLC system (Shimadzu Corporation, Kyoto, Japan), which was interfaced with a Sciex QTRAP 5500 mass spectrometer (Foster City, CA, USA).

Sample preparation included a liquid–liquid extraction of ARQ 501 and the internal standard dihydrotanshone (IS) from plasma. Briefly, 100 µL of a 700 ng/mL solution of IS in methanol:acetonitrile (3:1) was added to a 2-mL Eppendorf tube and spun to dryness on a CentriVap benchtop vacuum concentrator (Labconco, Kansas City, USA). To the dried tube, 25-µL aliquots of plasma, 25-µL of blank plasma, and 200-µL de-ionised water were added and vortex mixed. To the resultant solution, a 1-mL aliquot of methyl tert-butyl ether:ethyl acetate (1:1) was added. The mixture was shaken for 10 min at 2500 rpm on a VWR shaker. Samples were then centrifuged for 10 min at 4000 rpm at 4.0 °C. Subsequently, 750 µL of the resultant clear supernatant was transferred to a 2-mL Eppendorf tube and dried at 35 °C for 1.5 h using a CentriVap benchtop vacuum concentrator for 30 min. The dried sample was reconstituted with 300 µL of acetonitrile: water (1:1). A 1-µL sample was injected onto the analytical column (Kinetex C18, 1.7 µm, 30 × 2.1 mm), which was maintained at 40 ± 2 °C. Isocratic elution of ARQ 501 and internal standard was achieved using a flow rate of 0.75 mL/min, a mobile-phase composition of acetonitrile:water:formic acid (45:55:0.1), and 4-min run time. Detection of ARQ 501 and internal standard was carried out in multiple-reaction monitoring mode with positive polarity, by monitoring 242.9 > 186.9 for ARQ 501 and 278.9 > 205.2 for internal standard. Quadrupole Q1 and Q3 were set on unit resolution. The standard curve was linear (*r* > 0.995) over the concentration range of 10–2000 ng/mL.

The analytical data obtained were processed by the Analyst software™ (version 1.6.2). The PK parameters of ARQ 501 were estimated using the Phoenix WinNonLin (Certara USA, Clayton, MO, USA) software package.

### Correlative studies

Methods for correlative studies, including NQO1 polymorphisms and tumour NQO1 expression, are provided in Supplemental Materials.

### Statistical analysis

All patients receiving at least one dose of ARQ 761 were considered evaluable for safety analyses. Patients experiencing a DLT or those who received all scheduled doses of ARQ 761 within the DLT window were considered evaluable for DLT assessment. Patients who received at least a full dosing regimen of ARQ 761 within the DLT window and had at least one disease assessment following the initiation of therapy were considered evaluable for response.

Summary statistics for patient characteristics were reported. Adverse events were summarised in tables and presented by dose level, seriousness, severity, and relatedness. We determined the rate of disease control (partial response or stable disease) and 95% confidence intervals (CIs). We separated patients into NQO1-low and -high subgroups according to NQO1 IHC *H*-score using a data-driven approach to define an objective cutoff value by using model-based clustering method implemented by “mclust” package version 4.4 for R.^21^ Progression-free survival (PFS) time was calculated from the date of first dose until disease progression was detected. PFS curves were estimated using the product-limit method of Kaplan–Meier with the log-rank test. We assessed the association between pharmacodynamic end points, PKs, toxicity, and clinical outcomes using Pearson correlation, linear regression, and Cox proportional hazards.

## Results

### Patient characteristics and treatment exposure

A total of 78 patients had tumour tissue successfully tested for NQO1 expression. Tumour types and NQO1 expression levels are listed in Supplemental Table 1. This cohort included 42 patients who were accrued to the treatment study. Reasons that the other 36 patients were not enrolled to the treatment study included the following: (a) found to be ineligible; (b) did not need a new treatment initiated before the study ended; (c) no slot available; (d) clinician/patient preference; and (e) tumour tissue was NQO1-negative (an exclusion factor only after institution of the enrolment biomarker). Baseline patient characteristics are presented in Table 1. Initially, we treated 5 patients at the 195 mg/m^2^ weekly 1-h infusion schedule and 4 patients at the 390 mg/m^2^ 1-h infusion weekly schedule. We noted more frequent and more profound anaemia in the 390 mg/m^2^ cohort. In a single patient treated at the 390 mg/m^2^ dose level, we noted evidence of early recovery from haemolysis (despite ongoing weekly dosing) when the infusion duration was prolonged from 1 to 2 h due to a suspected mild infusion-related reaction. Accordingly, we modified the treatment schedule to a 2-h infusion administered either QOW or the first 2 out of 3 weeks. At any given dose level, the infusion duration could be prolonged to 3 h (associated with a lower *C*_max_) if the MTD was exceeded, rather than dose de-escalation.

**Table 1:** Baseline characteristics of patients receiving ARQ 761 treatment

Characteristic	Number (%) or median (range)
Total patients	42
Age (years)	65 (37–86)
Sex	
Male	25 (60)
Female	17 (40)
Race	
White	36 (86)
African-American	4 (10)
Asian	1 (2)
Other	1 (2)
Primary tumour site	
Lung	17 (40)
Colorectal	5 (12)
Bladder	4 (10)
Pancreas	4 (10)
Breast	2 (5)
Bile duct	2 (5)
Stomach	2 (5)
Other^a^	6 (14)
Number of lines of prior therapy	4 (1–12)

^a^One each of oesophageal, larynx, melanoma, Merkel cell, skin squamous cell, and thymus

Overall, 10 patients were treated at the 195 mg/m^2^ level (all weekly dosing; five received 1-h infusions; five received 2-h infusions), 17 patients at the 390 mg/m^2^ level (three 1-h infusions weekly; four 2-h infusions weekly; three 2-h infusions QOW; seven 2-h infusions 2 out of every 3 weeks), 11 patients at the 450 mg/m^2^ level (five 2-h infusions QOW, and six 2-h infusions 2 out of every 3 weeks), and 4 patients at the 550 mg/m^2^ level (one 2-h infusion 2 out of every 3 weeks, one 2-h infusion QOW, two 3-h infusions QOW) (Supplemental Fig. 1). Across all dose levels, the median number of treatment cycles was 2 (range 1–6). After analysis of the first 20 patients with available efficacy and NQO1 biomarker data, which occurred after accrual of the first 34 patients, enrolment was restricted to those patients with NQO1-positive tumours (defined as *H*-score ≥ 200). Study accrual was stopped after six additional patients were enrolled because, even in this enriched subset of patients, single-agent activity of ARQ 761 appeared modest and further clinical development would focus on combination regimens. Reasons for treatment discontinuation included disease progression (*n* = 35), DLT (*n* = 1), and non-DLT toxicities (*n* = 6). One patient died while on study, which was considered related to the underlying cancer and unrelated to study drug or procedure.

### Toxicity

Table 2 lists treatment-related adverse events experienced by ≥10% of patients according to grade and dose cohort. The principal treatment-related toxicities were anaemia, haemolysis, and hypoxia (representing possible methemoglobinaemia). Supplemental Table 2 lists principal toxicities according to grade, dose level, schedule, and duration. Across all dose levels, anaemia occurred in approximately 80% of patients (14% grade 3) and generally had characteristics consistent with a haemolysis mechanism (increase in reticulocyte count, decrease in haptoglobin level). As anticipated, a less frequent infusion schedule appeared to be better tolerated, with lower rates of fatigue and high-grade anaemia in the 2/3 weeks and QOW groups than the weekly group (Supplemental Table 2), presumably due to more opportunity for recovery from treatment-related anaemia events. Other lineages (white blood cells, platelets) were essentially unaffected, making myelosuppression unlikely. Haemolytic anaemia did not appear to be immune-mediated (Coomb’s test negative) and responded to transfusions. In no cases was haemolytic anaemia associated with renal dysfunction, which can arise in severe cases from renal tubular obstruction by free plasma haemoglobin. In general, we also observed improved tolerability with longer infusion time.

**Table 2:** Treatment-related toxicities occurring in ≥10% of patients (*N*, %)

Dose	195 mg/m^2^		390 mg/m^2^		450 mg/m^2^		550 mg/m^2^		Total	
*N*	10		17		11		4		42	
	Grade 1–2	Grade 3–4	Grade 1–2	Grade 3–4	Grade 1–2	Grade 3–4	Grade 1–2	Grade 3–4	Grade 1–2	Grade 3–4
Anaemia	8 (80)	2 (20)	14 (82)	3 (18)	11 (100)	4 (36)	3 (75)	1 (25)	36 (86)	10 (24)
Fatigue	2 (20)	0 (0)	7 (41)	1 (6)	6 (55)	0 (0)	2 (50)	1 (25)	17 (40)	2 (2)
Hypoxia	0 (0)	2 (20)	1 (6)	5 (29)	2 (18)	1 (9)	0 (0)	3 (75)	3 (7)	11 (26)
Vomiting	1 (10)	1 (10)	2 (12)	1 (6)	0 (0)	0 (0)	2 (50)	0 (0)	5 (12)	2 (5)
Nausea	1 (10)	1 (10)	1 (6)	0 (0)	1 (9)	0 (0)	3 (75)	0 (0)	6 (14)	1 (2)

Although we had hypothesised that the far lower concentration of HPβCD required for ARQ 761 formulation would result in less haemolytic anaemia than was seen with ARQ 501, once dose was escalated to 390 mg/m^2^, the prevalence and severity of this toxicity became apparent. To characterise further, we sought to obtain haemolysis parameters at the time of ≥grade 3 anaemia (Hgb < 8.0 g/dL) (Supplemental Table 3). Unfortunately, many of these cases have missing data because (a) we either had not started collecting the data by that point or (b) the anaemia event occurred in a setting other than our clinical trials unit. For those cases with available data at the time of grade 3 anaemia, we observed a wide range of haemolysis parameters, including bilirubin (0.4–2.0 mg/dL), LDH (165–560 units/L), haptoglobin (155–408 mg/dL), and reticulocytes (2.9–8.6%).

Transient decrease in peripheral oxygen saturation (a surrogate marker of possible methemoglobinaemia) was noted in cases at all dose levels. At the 195 mg/m^2^ dose level, oxygen saturation decreased 4–5% during infusion, with recovery starting upon infusion completion and levels usually returning to baseline within 2–3 h. Associated symptoms were rare. At higher dose levels, oxygen saturation levels decreased 10–12% during infusion and in some cases required 24 h to recover. In seven cases, arterial blood gas was obtained. In all of these cases, PaO_2_ was normal at the time of peripheral desaturation, consistent with possible methemoglobinaemia. At higher dose levels, clinical features suggestive of possible methemoglobinaemia were noted transiently, including dizziness and mild nausea. No cases featured cyanosis or organ dysfunction. Patients did not report dyspnoea, further supporting the observation that the peripheral oxygen desaturation did not represent true hypoxemia. These apparent cases of methemoglobinaemia were not associated with organ dysfunction. Methemoglobin levels were determined in seven cases. Despite a clinical picture highly suggestive of methemoglobinaemia, the results were normal, ranging from 0.2% to 0.8% (laboratory reference range 0.4–1.5%).

A total of five DLTs occurred at the following levels: 390 mg/m^2^ weekly (*N* = 3), 450 mg/m^2^ 2/3 weeks (*N* = 1), and 550 mg/m^2^ 2/3 weeks (*N* = 1). All DLTs reflected anaemia. Based on these events and consideration of the overall feasibility of long-term treatment, the MTD was considered 390 mg/m^2^ as a 2-h infusion administered every 2 weeks.

### Efficacy

**Fig. 1:**
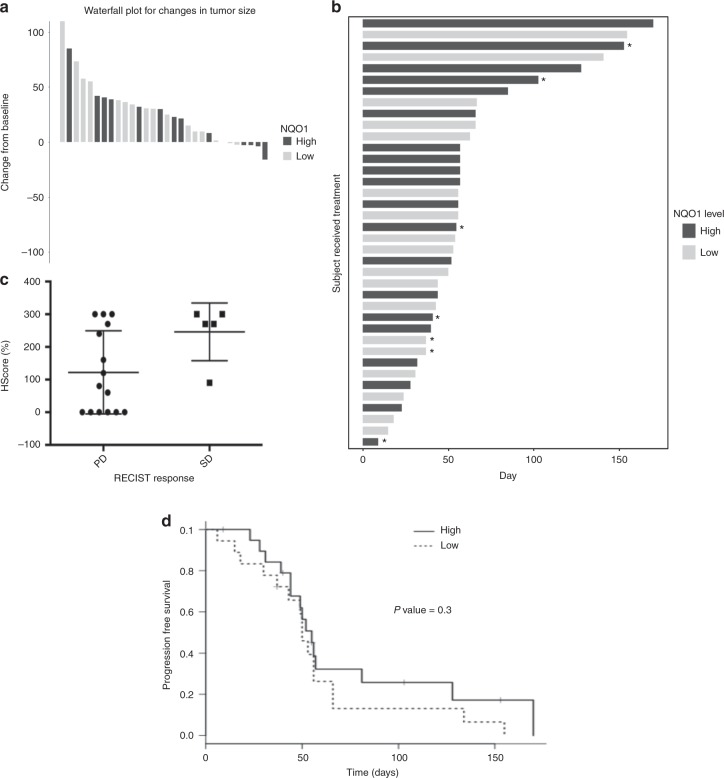
Efficacy of ARQ 761 according to tumour NQO1 expression. **a** Waterfall plot demonstrating best radiographic response in the overall study population. Dark bars indicate NQO1-high cases; light bars indicate NQO1-low cases. **b** Swimmer’s plot demonstrating time on therapy in the overall study population. Dark bars indicate NQO1-high cases; light bars indicate NQO1-low cases. Asterisks indicate cases for which treatment was discontinued for a reason other than disease progression. **c** Radiographic response among first 20 patients with both evaluable efficacy and tumour NQO1 expression data. NQO1 expression was higher among patients achieving stable disease compared to those with primary disease progression (*P* = 0.06). Of note, all six patients with NQO1-negative tumours had progressive disease. Based on these data, subsequent enrolment was limited to patients with NQO1-positive tumours (defined as *H*-score ≥ 200). PD progressive disease, RECIST, Response Evaluation Criteria in Solid Tumours, SD stable disease. **d** Progression-free survival according to tumour NQO1 expression. HR 0.68; 95% CI, 0.33–1.39; *P* = 0.3

**Fig. 2:**
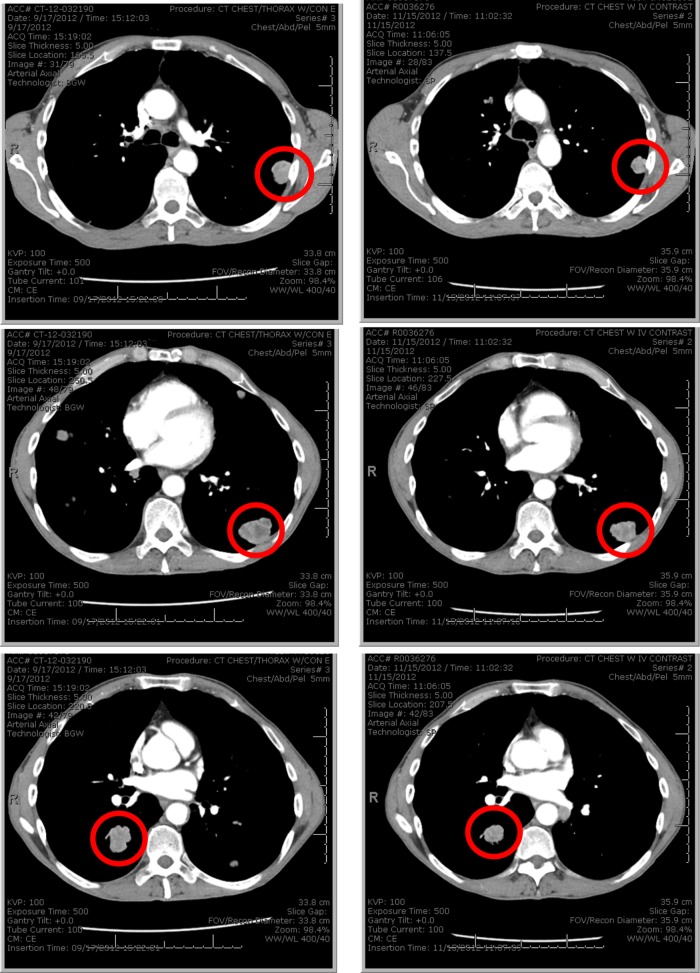
Example of minor radiographic response from ARQ 761. Patient had heavily pre-treated bladder cancer (five prior lines of therapy). Images show multiple pulmonary metastases that decreased in size after initiation of study treatment

Of the 32 patients evaluable for radiographic response, the best response was stable disease in 12 patients (disease control rate 38%) and progressive disease in 20 patients. Radiographic responses and duration of therapy are shown according to tumour NQO1 status in Fig. 1a, b. Minor radiographic response was noted in 6 patients, including 1 patient with heavily pre-treated (five prior lines of therapy) metastatic bladder cancer (NQO1 *H*-score 200) treated at dose level 195 mg/m^2^ who experienced shrinkage of multiple pulmonary metastases (Fig. 2). After analysis of 20 patients with evaluable response and available NQO1 expression level, we observed a near significant association (*P* = 0.06) between tumour NQO1 expression (*H*-score ≥ 200) and response (Fig. 1c). Of note, no NQO1-negative (*H*-score 0) cases (*N* = 6) achieved disease control. Based on these observations, the protocol was amended to limit enrolment to NQO1-positive cases (*H*-score ≥ 200), following which an additional 8 patients were treated. PFS according to NQO1 level for the entire study population is shown in Fig. 1d. Four-month PFS rate was 26% in NQO1-high tumours vs. 13% in NQO1-low tumours (*P* = 0.3).

### Pharmacokinetics

Across all dose and schedule cohorts, PK data were available for a total of 27 patients in the Dose 1 (first dose) group and a total of 17 patients in the Dose 4 (fourth dose) group. PKs of ARQ 761 were estimated using the concentration vs. time profiles of ARQ 501. The results of these PK determinations demonstrated high levels of patient variability. PKs were generally characterised by a rapid initial distribution and a slower terminal elimination; therefore, a two-compartment model was used to estimate the PKs.

The determined PK parameters for Dose 1 are presented in Table 3A and Dose 4 are presented in Table 3B. The use of a 2-h vs. 1-h infusion in Dose 1 resulted in a lower observed *C*_max_, which correlated well with the better tolerability of the 2-h infusions (Supplemental Table 2). For instance, in the 195 mg/m^2^ weekly dose cohort, the rate of anaemia was 100% (40% grade 3–4) with a 1-h infusion, compared to 60% (0% grade 3–4) with a 2-h infusion. The observed high variability of the area under the receiver operating characteristic curve (AUC) makes statistically significant clinical correlations difficult; however, the AUC did generally increase with increased dose. The half-life as measured as the half-life of elimination from the central compartment of the two-compartment model was fairly consistent among the dose levels and dosing regimen. PKs for Dose 1 vs. Dose 4 demonstrated that ARQ 501 does not accumulate to any appreciable extent using this dosing regimen. Within specific dose levels, a potential relationship between certain PK parameters and clinical effects was observed. These relationships included a trend toward correlation between Vss = apparent volume of distribution at steady state and PFS and a correlation between AUC and disease control (*P* = 0.03). There were no PK/pharmacodynamic relationships determined because pharmacodynamic end points were not included in the study.

**Table 3A:** Dose 1 pharmacokinetic parameters of plasma β-lapachone following intravenous administration of ARQ 761

Dose (mg/m^2^)	Infusion time	*N*	AUC_(0–last)_ (ng × h/mL)	*C*_max_ (ng/mL)	Half-life (h)^a^
195	1 h	4	10,463 ± 6103	1294 ± 258	1.8 ± 1.0
195	2 h	3	23,369 ± 15,1287	947 ± 233	3.1 ± 2.0
390	1 h	4	23,910 ± 20,745	2418 ± 705	1.2 ± 0.9
390	2 h	13	33,150 ± 27,468	1798 ± 738	2.6 ± 1.5
450	2 h	10	21,276 ± 13,323	1652 ± 442	1.5 ± 1.4

^a^Half-life of elimination from the central compartment of the two-compartment model

**Table 3B:** Dose 4 pharmacokinetic parameters of plasma β-lapachone following intravenous administration of ARQ 761

Dose (mg/m^2^)	Infusion time	*N*	AUC_(0–last)_ (ng × h/mL)	*C*_max_ (ng/mL)	Half-life (h)^a^
195	1 h	3	30,698 ± 9152	1540 ± 464	4.7 ± 1.7
195	2 h	4	32,007 ± 16,793	968 ± 63	3.5 ± 1.9
390	1 h	2	20,374 ± 7775	2785 ± 754	1.6 ± 0.3
390	2 h	3	41,537 ± 33,968	3215 ± 1658	2.5 ± 2.0
450	2 h	9	26,699 ± 14,251	2321 ± 1339	2.4 ± 2.4

^a^Half-life of elimination from the central compartment of the two-compartment model

### Biomarker studies

**Fig. 3:**
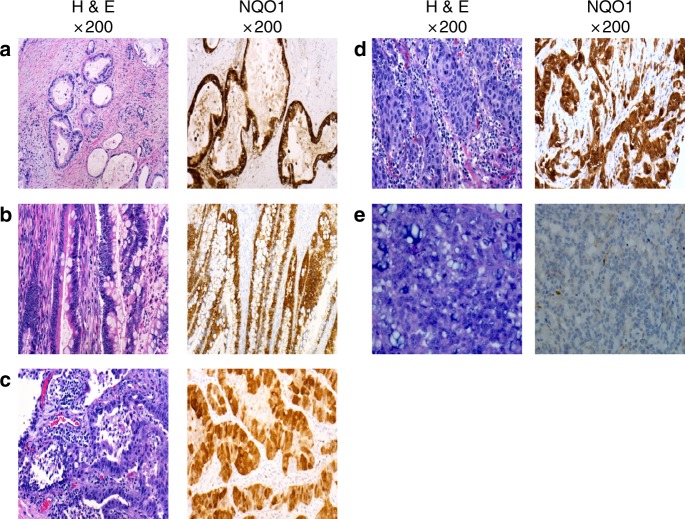
NQO1 IHC staining. **a** Moderately differentiated pancreatic adenocarcinoma, 3+ NQO1 staining, *H*-score 300. **b** Moderately differentiated colonic adenocarcinoma, 3+ NQO1 staining, *H*-score 250. **c** Lung adenocarcinoma, 3+ NQO1 staining, *H*-score 290. **d** High-grade invasive ductal breast carcinoma, 3+ NQO1 staining, *H*-score 300. **e** Prostate adenocarcinoma, 0+ NQO1 staining, *H*-score 0

We successfully determined tumour NQO1 expression by IHC in all enrolled cases (Fig. 3). Once enrolment was limited to NQO1-positive cases, we developed a CLIA-certified IHC assay with a turnaround time of ≤72 h permitting real-time assessment of potential study candidates. Germline NQO1 polymorphisms were assessed in all enrolled patients. All cases were NQO1*1.

## Discussion

This study is a first-in-human phase 1 trial of ARQ 761, a synthetic analogue of the NQO1-bioactivatable drug β-lap that is administered intravenously through central access. In this trial, we observed haemolytic anaemia as a DLT, with an MTD and recommended phase 2 dose of 390 mg/m^2^ as a 2-h infusion QOW. At this level, patients also experienced largely asymptomatic possible methemoglobinaemia, manifest as transient decrease in peripheral oxygen saturation without a concomitant decrease in PaO_2_. As we had hypothesised, ARQ 761 appeared to exert antitumour effects in an NQO1-dependent fashion. All efficacy parameters (response, PFS, time on study treatment) had either a statistically significant (or trend towards) association with tumour NQO1 expression. Although no patients experienced formal partial response by RECIST parameters, approximately 20% of cases had some evidence of radiographic shrinkage, including at the lowest dose level tested.

Compared to the previous formulation, ARQ 501, ARQ 761 requires only 5% the total HPβCD—the carrier molecule thought to be the aetiology of haemolysis in preclinical models. Nevertheless, as with ARQ 501, an apparent haemolytic anaemia emerged as the principal toxicity and DLT in the present trial. The characterisation of the observed anaemia is challenging. The known mechanism of β-lap, as well as the lack of leukopenia and thrombocytopenia in this trial, make bone marrow suppression unlikely. Instead, separate from direct effects of the HPβCD, ARQ 761-mediated changes in the redox state may account for this toxicity. While β-lap is a very good substrate for the two-electron oxidoreductase, NQO1, it is also an excellent substrate for one-electron oxidoreductases, such as the b5R enzyme.^1^ In particular, the toxic effects of β-lap may be due to its interactions with b5R, which is expressed in the blood of mammals, where b5R is responsible for reducing haemoglobin. In the presence β-lap, b5R fails to reduce methemaglobin and results in more one-electron oxidoreductions that further oxidise the haeme molecules, contributing to methemoglobinaemia. Subsequently, it is possible that methemoglobinaemia itself drove the haemolytic events, as has been reported with other therapeutic agents, such as rasburicase.^22^ Although possible methemoglobinaemia was clinically evident in approximately 30% of patients (and was suspected in essentially all patients treated at higher-dose levels), methemoglobin levels were not elevated. This phenomenon may reflect challenges in the performance of this assay, which is subject to variations according to timing and handling of sample, and may also be influenced by haemolysis in the specimen.^23^ Furthermore, in animal models, β-lap-induced methemoglobinaemia lasted only 5 min, so the event may be too transient for clinical detection.^24^ Without reliable methemoglobin levels in our study population for guidance, we consider the observed ARQ 761-associated possible methemoglobinaemia to be relatively mild given the transient nature of desaturation by pulse oximetry and the absence of cyanosis. Certainly, we have little suspicion that patients experienced life-threatening events (which occur at methemoglobin levels >70%). One possible approach to mitigating methemoglobinaemia is the administration of antioxidants. However, it is not known whether such a strategy would reduce therapeutic anticancer effects. In any case, although ARQ 761 does not appear to cause myelosuppression, the potential for overlapping anaemia toxicity will necessitate careful consideration when this agent is combined with cytotoxic therapies.

ARQ 761 PKs demonstrated high variability, which may in part reflect the small number of patients in many of the dosage/schedule/infusion time groups. Additionally, the PK profile of prodrugs is generally more variable when compared to the parent drug because of inter-patient differences in the rate of conversion from prodrug to parent drug (ARQ 501). This highly variable PK profile may complicate ARQ 761 administration; agents with high variability tend to have higher adverse event rates because they often cross the adverse event concentration threshold.

This study is the first human clinical trial study to suggest an association between β-lap efficacy and positive expression of NQO1 in tumours. Pharmacologically, NQO1 has a role distinct from typical “targets” of small molecules and monoclonal antibodies. Instead of driving cancer cell survival pathways that can be inhibited by targeted therapies (e.g. epidermal growth factor receptor, vascular endothelial growth factor receptor), NQO1 activates ARQ 761 to exert cytotoxic effects via oxidative stress. Consistent with earlier reports,^25–30^ we observed high rates and levels of NQO1 expression in NSCLC and gastrointestinal malignancies. In contrast, only one of the three neuroendocrine lung cancers expressed NQO1, consistent with earlier reports.^31,32^ As expected for Western populations, we identified no *2 or *3 NQO1 genotypic polymorphisms in this study cohort. Such variants could influence NQO1 tissue expression and activity, and they may need to be considered in clinical trials in East Asian groups, in which NQO1 polymorphisms occur in up to 13% of individuals.^33^

Although this phase 1 trial suggests that tumours with high expression of NQO1 by IHC may be associated with clinical benefit of ARQ 761 and other β-lap analogues, from this relatively small sample it is not clear that this assay or the selected cut point (*H*-score 200) represents the optimal enrollment biomarker for β-lap trials. Most of the tissue samples analysed for NQO1 expression came from archival specimens. It is possible that NQO1 expression may have changed during the intervening lines of therapy, as preclinical models have demonstrated that ionising radiation and multiple cytotoxic therapies may induce NQO1.^34,35^ Furthermore, biopsy specimens have limited tumour tissue that may not reflect potential intratumoural heterogeneity. Based on preclinical studies, we hypothesised that a combination of elevated NQO1 and low catalase (a detoxifying enzyme) in tumour specimens—reported as an elevated NQO1/catalase ratio—would provide the most meaningful predictor of efficacy.^36^ However, we were not able to reliably quantify tissue catalase expression in the available clinical samples. The observed PK parameters further complicate these considerations, as it appeared that plasma ARQ 761 levels may vary substantially within a given dose level and could be associated with clinical benefit. Unfortunately, to date, we have not developed a clinically available non-invasive pharmacodynamic biomarker for β-lap. Although this study did not include on-study biopsies, the ongoing trial of ARQ 761 in combination with gemcitabine plus Nab-paclitaxel (NCT02514031) does incorporate post-treatment biopsies. These will allow serial monitoring of intratumoural PARYlated PARP, which correlates with antitumour effects in preclinical models.^1,37^ Imaging of hyperpolarised glucose or pyruvate represents another potential pharmacodynamic biomarker.^30^ Finally, this single-arm phase 1 study does not itself provide evidence that NQO1 expression is predictive rather than prognostic. However, NQO1 expression has been associated with *worse* outcomes in multiple tumour types.^38–41^ Thus the trend towards improved outcomes in NQO1-high cases in this trial seems likely to reflect treatment effects.

In conclusion, ARQ 761 is a synthetic analogue and prodrug of the NQO1-bioactivatable drug β-lapachone. In this phase 1 trial, ARQ 761 demonstrated highly variable PKs and possible methemoglobinaemia and haemolytic anaemia emerged as the principal toxicities. Although we had hypothesised that its formulation would mitigate key adverse effects, the ARQ 761 MTD of 390 mg/m^2^ was identical to that of the earlier ARQ 501 compound. As monotherapy, ARQ 761 had only modest efficacy, resulting in minor radiographic responses in approximately 20% of patients but no partial responses. Consistent with preclinical models, this effect may be associated with tumours that have high NQO1 expression, but further biomarker development and PK profiling will be required to identify optimal populations for this treatment strategy. Recently, in vitro and in vivo studies have demonstrated synergistic effects when β-lap is combined with other treatments such as PARP inhibitors, ionising radiation, alkylating agents, glutamine metabolism inhibitors, and nicotinamide phosphoribosyltransferase inhibitors.^27,28,37,42,43^ Clinical evaluation of such combinations may be warranted, with careful attention to the potential for overlapping toxicity. Combination studies with chemotherapy (gemcitabine and Nab-paclitaxel for pancreatic cancer; NCT02514031) and olaparib (refractory advanced solid tumours; NCT03575078) are underway. Emerging alternative drug formulations, such as controlled release micelles and intratumoural polymer implants,^6,8,26^ may improve the tolerability of this treatment strategy. Alternatively, NQO1-bioactivatable drugs that are not good substrates for one-electron oxidoreductases (e.g. b5R)—including deoxynyboquinone and derivatives such as isobutyldeoxynyboquinone^24^—may mitigate the systemic redox changes that may underlie the key toxicities of ARQ 761.

## Electronic supplementary material

revised supp materials

Supplement Figure 1

## Data Availability

The data sets generated during and/or analysed during the current study are available from the corresponding author on reasonable request.
