# Supplementary material for: Phase 1 study of ARQ 761, a β-lapachone analogue that promotes NQO1-mediated programmed cancer cell necrosis
Source: Br J Cancer. 2018 Oct 15;119(8):928–36. doi: 10.1038/s41416-018-0278-4 (PMC6203852; doi:10.1038/s41416-018-0278-4)
Supplement: Supplementary file 1 — revised supp materials [file 41416_2018_278_MOESM1_ESM.docx]

**SUPPLEMENTARY MATERIALS**

**Phase 1 Study of ARQ 761, a β-lapachone analog that promotes NQO1-mediated programmed cancer cell necrosis**

David E. Gerber^1,2,3^*, M. Shaalan Beg^1,3^, Farjana Fattah^3^, Arthur E. Frankel^1,3^, Oluwatomilade Fatunde^3^, Yull Arriaga^1,3^, Jonathan E. Dowell^1,3^, Ajit Bisen^1^, Richard D. Leff^4^, Claudia C. Meek^4^, William C. Putnam^4^, Raja Reddy Kallem^4^, Indhumathy Subramaniyan^4^, Ying Dong^3^, Joyce Bolluyt^3^, Venetia Sarode^5^, Xin Luo^7^, Yang Xie^2,3,7^, Brian Schwartz^6^, David A. Boothman^3^

^1^Department of Internal Medicine (Division of Hematology-Oncology), University of Texas Southwestern Medical Center, Dallas, Texas; ^2^Department of Clinical Sciences, University of Texas Southwestern Medical Center, Dallas, Texas; ^3^Harold C. Simmons Comprehensive Cancer Center, University of Texas Southwestern Medical Center, Dallas, Texas; ^4^Texas Tech University Health Sciences Center School of Pharmacy, Dallas, Texas; ^5^Department of Pathology, University of Texas Southwestern Medical Center, Dallas, Texas; ^6^ArQule, Inc., Burlington, Massachusetts. ^7^Department of Bioinformatics, University of Texas Southwestern Medical Center, Dallas, Texas, USA

*** Corresponding Author:**

David E. Gerber, MD

Division of Hematology-Oncology

Harold C. Simmons Cancer Center

University of Texas Southwestern Medical Center

5323 Harry Hines Blvd.

Mail Code 8852

Dallas, Texas 75390-8852

Phone: 214-648-4180

Fax: 214-648-1955

E-mail: [david.gerber@utsouthwestern.edu](mailto:david.gerber@utsouthwestern.edu)

**Supplemental Figure Legends.**

**Supplemental Figure 1. Treatment cohorts and disposition.** DLT, dose limiting toxicity; NE, not evaluable; QOW, every other week

**Supplemental Table 1.** NQO1 H-scores among screened cases. Cases listed by tumor type in ascending order of H-score.

| **Tumor site** | **H-score** |
| --- | --- |
| *Gastrointestinal* | |
| Anus | 0 |
| Anus | 80 |
| Bile duct | 0 |
| Bile duct | 70 |
| Bile duct | 110 |
| Bile duct | 300 |
| Bile duct | 300 |
| Colon | 60 |
| Colon | 100 |
| Colon | 130 |
| Colon | 190 |
| Colon | 210 |
| Colon | 250 |
| Colon | 260 |
| Esophagus | 210 |
| Esophagus | 250 |
| Esophagus | 280 |
| Gallbladder | 0 |
| Gallbladder | 280 |
| Pancreas | 0 |
| Pancreas | 0 |
| Pancreas | 110 |
| Pancreas | 110 |
| Pancreas | 200 |
| Pancreas | 210 |
| Pancreas | 210 |
| Pancreas | 210 |
| Pancreas | 260 |
| Pancreas | 260 |
| Pancreas | 280 |
| Pancreas | 300 |
| Pancreas | 300 |
| Rectum (neuroendocrine) | 0 |
| Rectum | 40 |
| Rectum | 140 |
| Stomach | 250 |
| *Genitourinary* | |
| Bladder | 0 |
| Bladder | 80 |
| Bladder | 180 |
| Bladder | 200 |
| Bladder | 240 |
| Bladder | 250 |
| Kidney | 170 |
| Prostate | 120 |
| Prostate | 300 |
| *Upper aerodigestive* | |
| Head and neck | 125 |
| Head and neck | 260 |
| Head and neck | 300 |
| Lung (neuroendocrine) | 0 |
| Lung | 0 |
| Lung | 0 |
| Lung | 0 |
| Lung (neuroendocrine) | 10 |
| Lung | 20 |
| Lung | 50 |
| Lung | 70 |
| Lung | 100 |
| Lung | 125 |
| Lung | 210 |
| Lung | 220 |
| Lung | 230 |
| Lung | 230 |
| Lung | 240 |
| Lung (neuroendocrine) | 290 |
| Lung | 290 |
| Lung | 290 |
| Lung | 300 |
| Lung | 300 |
| Lung | 300 |
| Pleura | 0 |
| Pleura | 210 |
| Thymus | 0 |
| Thymus | 0 |
| *Other* | |
| Breast | 0 |
| Skin (squamous) | 0 |
| Merkel cell | 0 |
| Adrenal | 140 |
| Endometrium | 200 |

**Supplemental Table 2.** Principal toxicities according to dose level, infusion duration, and infusion schedule.

| **Grade 1-2** |  |  |  |  |  |  |  |  |  |  |  |
| --- | --- | --- | --- | --- | --- | --- | --- | --- | --- | --- | --- |
| **Dose (mg/m^2^)** | **195** | | **390** | | | | **450** | | **550** | | |
| **Frequency** | Weekly | Weekly | Weekly | Weekly | 2/3 wks | QOW | 2/3 wks | QOW | 2/3 wks | QOW | QOW |
| **Duration (h)** | 1 | 2 | 1 | 2 | 2 | 2 | 2 | 2 | 2 | 2 | 3 |
| **N** | 5 | 5 | 4 | 3 | 7 | 3 | 6 | 5 | 1 | 1 | 2 |
| **Anemia** | 5 (100) | 3 (60) | 3 (75) | 2 (67) | 6 (86) | 3 (100) | 6 (100) | 5 (100) | 1 (100) | 1 (100) | 1 (50) |
| **Fatigue** | 1 (20) | 1 (20) | 4 (100) | 2 (67) | 2 (29) | 1 (33) | 5 (83) | 1 (20) | 0 (0) | 0 (0) | 1 (50) |
| **Hypoxia** | 0 (0) | 0 (0) | 1 (25) | 0 (0) | 1 (14) | 0 (0) | 1 (17) | 1 (20) | 0 (0) | 0 (0) | 0 (0) |
| **Vomiting** | 1 (20) | 1 (20) | 1 (25) | 1 (33) | 1 (14) | 0 (0) | 0 (0) | 0 (0) | 1 (100) | 1 (100) | 0 (0) |
| **Nausea** | 0 (0) | 2 (40) | 0 (0) | 0 (0) | 1 (14) | 0 (0) | 1 (17) | 0 (0) | 1 (100) | 1 (100) | 1 (50) |
|  |  |  |  |  |  |  |  |  |  |  |  |
| **Grade 3-4** |  |  |  |  |  |  |  |  |  |  |  |
| **Dose**  **(mg/m^2^)** | **195** | | **390** | | | | **450** | | **550** | | |
| **Frequency** | Weekly | Weekly | Weekly | Weekly | 2/3 wks | QOW | 2/3 wks | QOW | 2/3 wks | QOW | QOW |
| **Duration (h)** | 1 | 2 | 1 | 2 | 2 | 2 | 2 | 2 | 2 | 2 | 3 |
| **N** | 5 | 5 | 4 | 3 | 7 | 3 | 6 | 5 | 1 | 1 | 2 |
| **Anemia** | 2 (40) | 0 (0) | 0 (0) | 1 (33) | 2 (29) | 0 (0) | 2 (33) | 2 (40) | 0 (0) | 0 (0) | 1 (50) |
| **Fatigue** | 1 (20) | 0 (0) | 1 (33) | 0 (0) | 0 (0) | 0 (0) | 0 (0) | 0 | 1 (100) | 0 (0) | 0 (0) |
| **Hypoxia** | 0 (0) | 2 (40) | 2 (67) | 2 (67) | 2 (29) | 1 (33) | 0 (0) | 1 (20) | 1 (100) | 1 (100) | 2 (100) |
| **Vomiting** | 0 (0) | 1 (20) | 0 (0) | 1 (33) | 0 (0) | 0 (0) | 0 (0) | 0 (0) | 0 (0) | 0 (0) | 0 (0) |
| **Nausea** | 0 (0) | 1 (20) | 0 (0) | 0 (0) | 0 (0) | 0 (0) | 0 (0) | 0 (0) | 0 (0) | 0 (0) | 0 (0) |

**Supplemental Table 3.** Nadir red blood cell and hemolysis parameters in patients who developed ≥grade 3 anemia (Hgb <8.0 g/dL)

| **Dose Level** | **Infusion schedule (infusion duration)** | **Nadir time-point** | **Nadir Hgb (g/dL)/Hct (%)** | **LDH (units/L)** | **Tbili (mg/dL)** | **Haptoglobin (mg/dL)** | **Reticulocytes (%)** |
| --- | --- | --- | --- | --- | --- | --- | --- |
| 195 | Weekly (1 hr) | C1D8 | 7.8/22.1 | 216 | 0.5 | N/A | N/A |
| 195 | Weekly (1 hr) | C2D15 | 7.7/23.2 | 470 | N/A | 172 | N/A |
| 390 | Weekly (1 hr) | C1D5 | 6.3/18.1 | N/A | 2.0 | N/A | N/A |
| 390 | 2/3 weeks (2 hrs) | C2D8 | 7.7/25.2 | 560 | 1.1 | 155 | 8.6 |
| 390 | 2/3 weeks (2 hrs) | C1D5 | 7.7/24.9 | N/A | N/A | N/A | N/A |
| 450 | 2/3 weeks (2 hrs) | C3D1 | 7.4/21.8 | 165 | 0.2 | 195 | 6.9 |
| 450 | QOW (2 hrs) | C1D15 | 6.3/20.8 | 541 | 1.1 | 408 | 3.7 |
| 450 | QOW (2 hrs) | C2D22 | 7.9/24.6 | 204 | 0.4 | 145 | 3 |
| 450 | 2/3 weeks (2 hrs) | C2D9 | 7.7/25.8 | 357 | 0.7 | 173 | 6.5 |
| 550 | QOW (3 hrs) | C1D20 | 7.2/23.7 | 434 | 0.4 | N/A | 2.9 |

C, cycle; D, day; Hgb, Hemoglobin; Hematocrit, Hct; N/A, not available; QOW, every other week

**Supplemental Text:**

**Methods**

**Correlative Studies**

Biospecimens for correlative studies included 10 unstained slides or a paraffin-embedded tissue block from an archived tumor specimen, as well as pre- and post-infusion blood samples from infusions 1 and 4. Tumor tissue was tested for NQO1 and catalase expression by immunohistochemistry as previously described.([1](#_ENREF_1), [2](#_ENREF_2)) Polymerase chain reaction–Restriction fragment length polymorphism (PCR-RFLP) analyses of the NQO1 gene([3](#_ENREF_3))for *2 and *3 polymorphisms were carried out using PCR amplification with the primer set of 5′-TCCTCAGAGTGGCATTCTGC-3′ and 5′-TCTCCTCATCCTGTACCTCT-3′ (for *2) or 5′-TCAAGTTGGCTGACCAAGGACA-3′ and 5′-CCTGCATCAGTACAGACCAC-3′ (for *3). The thermal cycling conditions were as follows: initial denaturation for 10 min at 94 °C, 40 cycles of denaturation at 94 °C for 1 min, annealing at 55 °C for 1 min, elongation at 72 °C for 1 min, and final primer extension at 72 °C for 5 min. The amplified PCR products were then digested at 37 °C for 2 hours with either Hinf-1 (for *NQO1**2) or Msp-1 (for *NQO1**3) and analyzed using electrophoresis on a 1.0% agarose gel.

All tissue-based correlative studies were performed in the Clinical Laboratory Improvement Amendments (CLIA)- and College of American Pathologists (CAP)-certified laboratory of the Department of Pathology, UT Southwestern Medical Center. Formalin-fixed paraffin embedded tumor tissue was processed using a standard protocol. Four to five micron sections of the tumor were cut, and one section was stained with hematoxylin and eosin (H&E) to determine tumor adequacy. NQO1 immunostaining (clone AB34173, ABCAM, MA, dilution of 1:200) was performed by standardized automated technique using the Ultra Benchmark system (Ventana Medical Systems, Roche). Briefly, tissue sections were deparaffinized followed by cell conditioning (antigen retrieval), which hydrolyzed covalent bonds formed by formalin in tissue. We used the ultraView DAB Detection Kit, which contains a cocktail of enzyme-labeled secondary antibodies that locate the bound primary antibody. Endogenous peroxidase was blocked by hydrogen peroxide, included in the ultraView DAB Detection Kit. The complex was then visualized with hydrogen peroxide substrate and 3,3'-diaminobenzidine tetra hydrochloride (DAB) chromogen, which produces a dark brown precipitate. Positive and negative controls were placed on the same slide as the tumor tissue (same slide control) to ensure that the antibody and other reagents were dispensed in the same fashion as the tumor tissue.

A semi-quantitative assessment of NQO1 expression (cytoplasmic and nuclear positive staining) was performed using the Histoscore method. The H-score is evaluated based on the intensity of the staining graded from 0 to 3+ and percentage (%) of positive staining cells according to the following formula: (1 x % of 1+ cells) + (2 x % of 2+ cells) + (3 x % of 3+ cells) = H-Score (range from 0 to 300). An H-score of ≥200 was our cut off for positive NQO1 expression in this study. Based on preclinical models,([4](#_ENREF_4)) we initially hypothesized that ARQ 761 efficacy would be associated with tumor NQO1 expression and inversely proportional to tumor catalase expression. However, we were not able to perform catalase staining successfully.

**References**

1. Siegel D, Ross D. Immunodetection of NAD(P)H:quinone oxidoreductase 1 (NQO1) in human tissues. Free Radic Biol Med. 2000 Aug;29(3-4):246-53. PubMed PMID: 11035253. Epub 2000/10/18. eng.

2. Lakari E, Paakko P, Pietarinen-Runtti P, Kinnula VL. Manganese superoxide dismutase and catalase are coordinately expressed in the alveolar region in chronic interstitial pneumonias and granulomatous diseases of the lung. Am J Respir Crit Care Med. 2000 Feb;161(2 Pt 1):615-21. PubMed PMID: 10673208. Epub 2000/02/15. eng.

3. Phillips RM, Basu S, Brown JE, Flannigan GM, Loadman PM, Martin SW, et al. Detection of (NAD(P)H:Quinone oxidoreductase-1, EC 1.6.99.2) 609C-->T and 465C-->T polymorphisms in formalin-fixed, paraffin-embedded human tumour tissue using PCR-RFLP. Int J Oncol. 2004 Apr;24(4):1005-10. PubMed PMID: 15010841. Epub 2004/03/11. eng.

4. Pink JJ, Planchon SM, Tagliarino C, Varnes ME, Siegel D, Boothman DA. NAD(P)H:Quinone oxidoreductase activity is the principal determinant of beta-lapachone cytotoxicity. J Biol Chem. 2000 Feb 25;275(8):5416-24. PubMed PMID: 10681517. Epub 2000/02/22. eng.
