# Supplementary figures and images for: Phase 1 study of ARQ 761, a β-lapachone analogue that promotes NQO1-mediated programmed cancer cell necrosis
Source: Br J Cancer. 2018 Oct 15;119(8):928–36. doi: 10.1038/s41416-018-0278-4 (PMC6203852; doi:10.1038/s41416-018-0278-4)

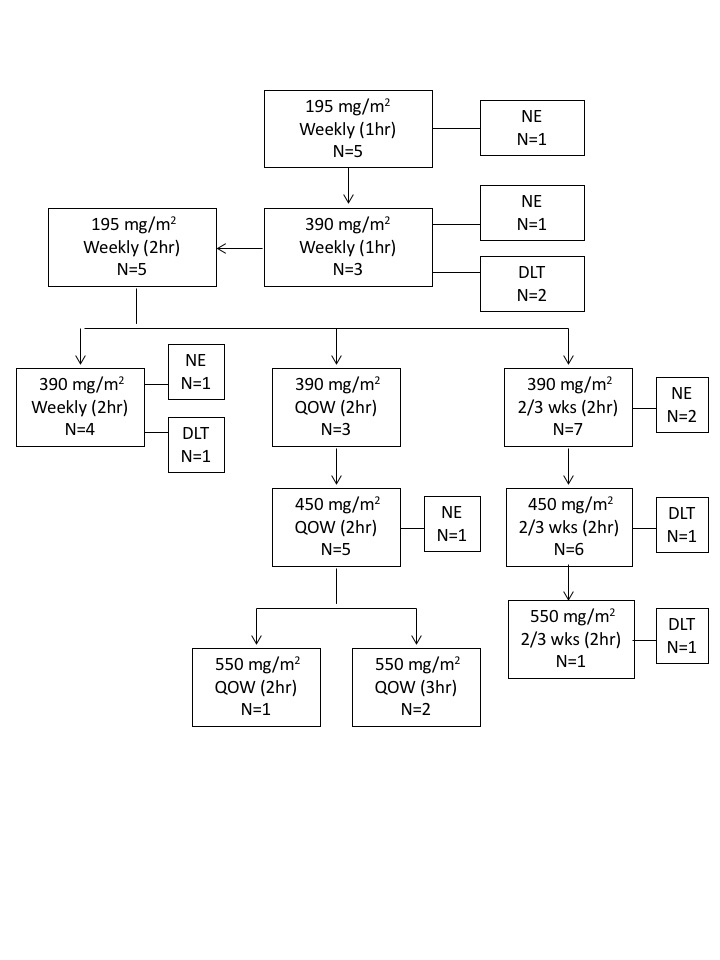

Supplement: Supplementary file 2 — Supplement Figure 1 [file 41416_2018_278_MOESM2_ESM.jpg]
